# Supplementary material for: Nucleolar Stress Response via Ribosomal Protein L11 Regulates Topoisomerase Inhibitor Sensitivity of P53-Intact Cancers
Source: Int J Mol Sci. 2022 Dec 15;23(24):15986. doi: 10.3390/ijms232415986 (PMC9784028; doi:10.3390/ijms232415986)
Supplement: Supplementary file 1 [file ijms-23-15986-s001.zip › ijms-2027907-supplementary.pdf]

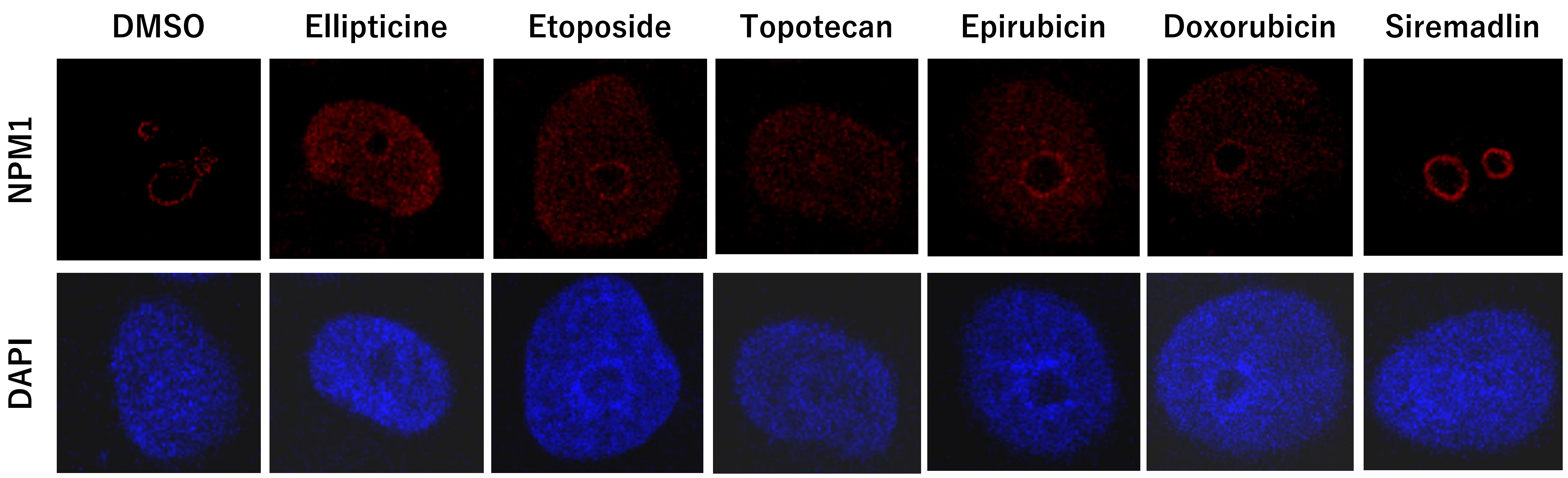

Figure S1. Alteration of nucleolar morphology by treatment with the topoisomerase inhibitors. JMU-RTK-2 cells were treated with 3 μM Ellipticine, 30 μM Etoposide, 1 μM Topotecan, 1 μM Siremadlin, 0.3 μM Epirubicin, or 0.1 μM Doxorubicin. After 24 h of culture, cells were immunostained with antibody for nucleophosmin (NPM1), followed by nuclear staining with DAPI.

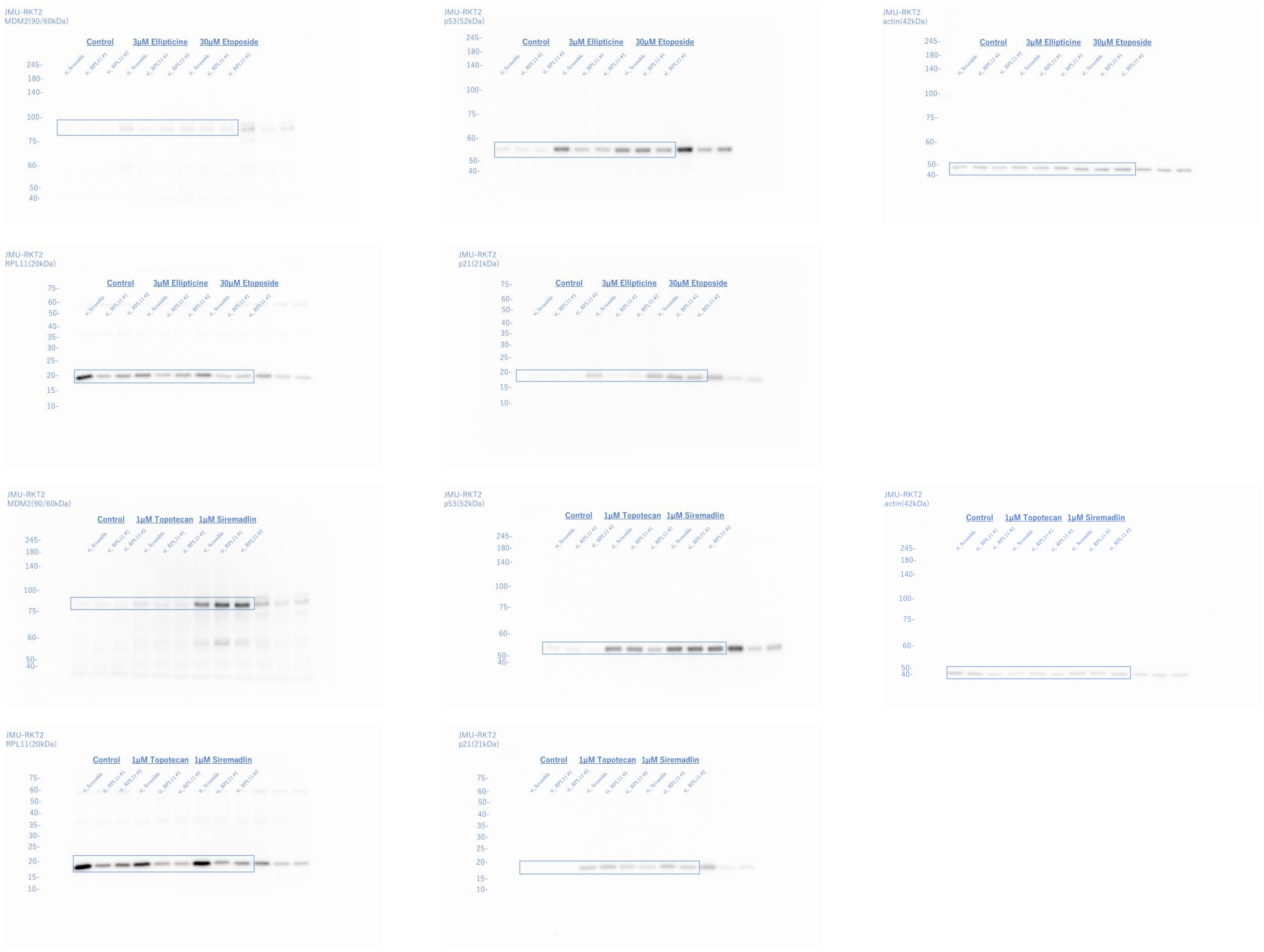

Figure S2. whole blot images of Figure 3.

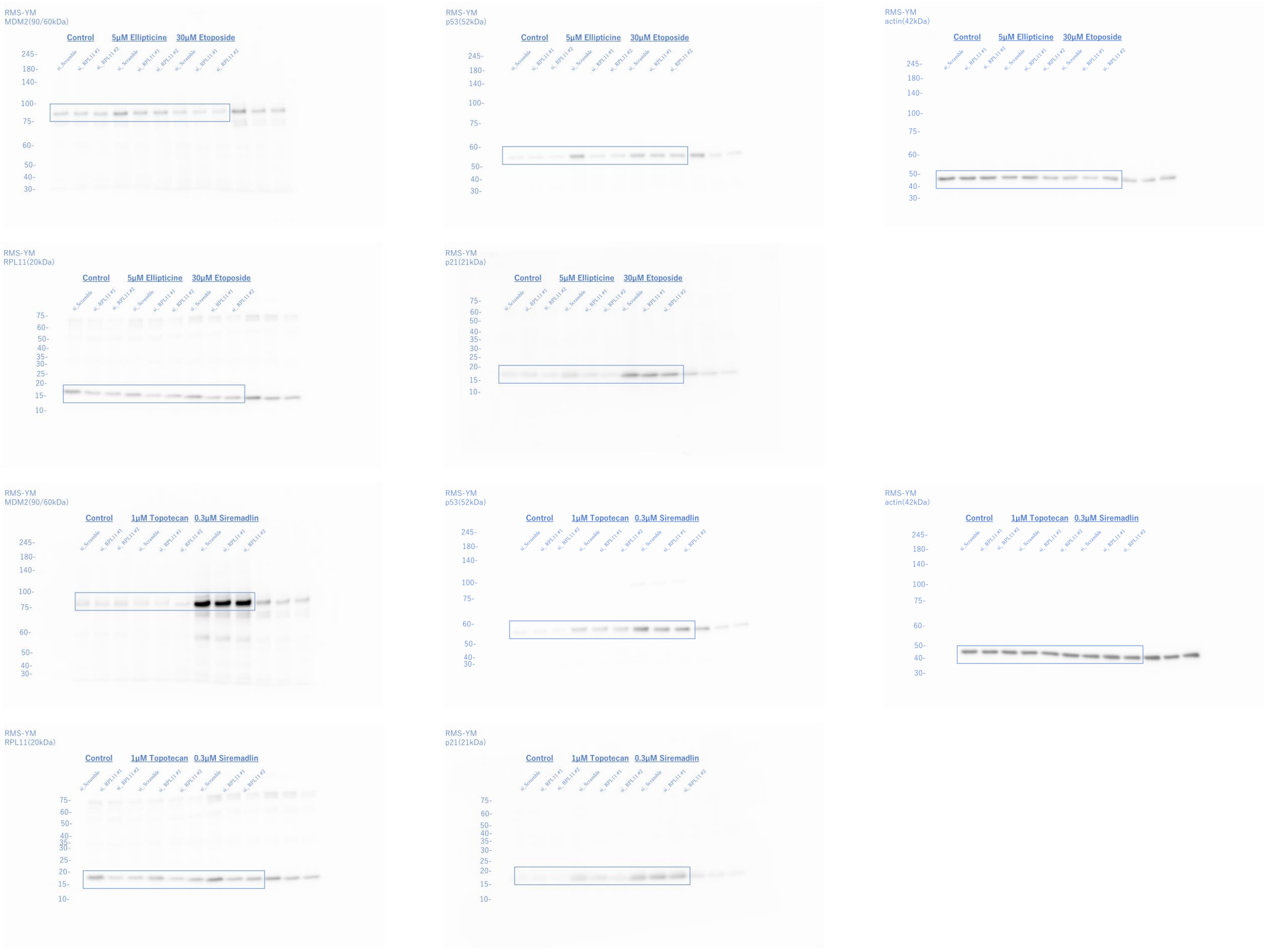

Figure S3. whole blot images of Figure 4.

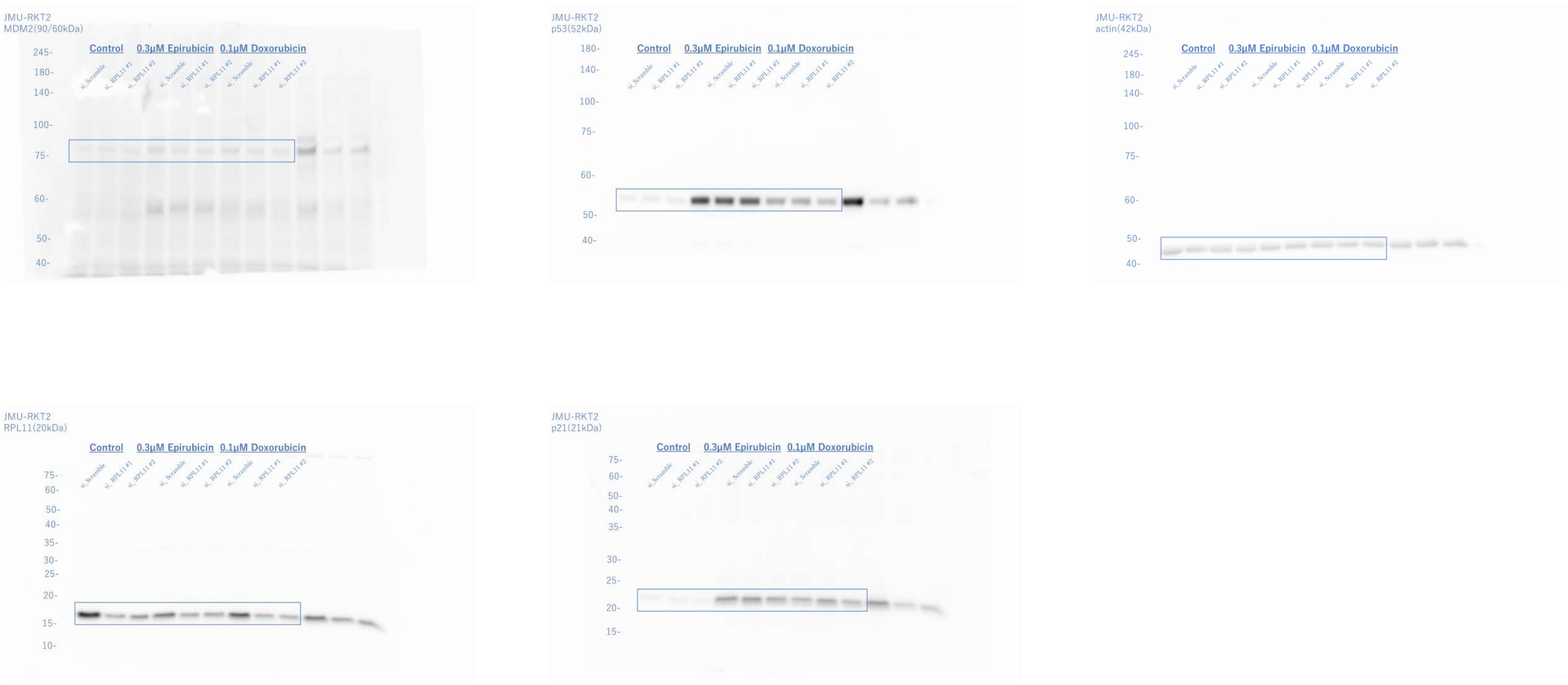

Figure S4. whole blot images of Figure 5C.
